# The combination of living *Bifidobacterium, Lactobacillus*, and *Streptococcus* improves social ranking and relieves anxiety‐like behaviors in competitive mice in a social dominance tube test

**DOI:** 10.1002/brb3.2453

**Published:** 2021-12-08

**Authors:** Jianping Xie, Yun Yuan, Heng Tan, Yufan Bai, Qingyue Zheng, Lin Mao, Yushan Wu, Ling Wang, Wenhui Da, Qingyan Ye, Suting Zhang, Jing Wang, Wenyao Yin, Yujing Bian, Wenjie Ma, Lanchun Zhang, Rongping Zhang, Haofei Yu, Ying Guo

**Affiliations:** ^1^ School of Basic Medical Sciences Kunming Medical University Kunming Yunnan P. R. China; ^2^ School of Pharmaceutical Science, Department of Zoology & Yunnan Key Laboratory of Pharmacology for Natural Products Kunming Medical University Kunming Yunnan P. R. China; ^3^ Library Yunnan Minzu University Kunming Yunnan P. R. China; ^4^ School of Chinese Materia Medica and Yunnan Key Laboratory of Southern Medicinal Resources Yunnan University of Traditional Chinese Medicine Kunming Yunnan P. R. China

**Keywords:** anxiety, Bifidobacterium, Lactobacillus, ranking score, Streptococcus

## Abstract

**Introduction:**

Social rank has a profound influence on the behavior and health of humans and animals.

**Methods:**

To explore the effect of a combination of living *Bifidobacterium, Lactobacillus* and *Streptococcus* (CLB) on anxiety‐ and depression‐like behaviors and social rank, mice were subjected to a social dominance tube test (SDTT). The behaviors, rank, gut microbiota, and expression of inflammatory cytokines and brain‐derived neurotrophic factor (BDNF) in the hippocampus were measured.

**Results:**

The results indicated that CLB improved the SDTT ranking score of the losers and alleviated anxiety‐like behaviors of the winners. CLB decreased the level of *Desulfovibrio* and augmented the level of *Mollicutes* in the feces, increased BDNF content, and reduced the level of tumor necrosis factor‐α in the hippocampus.

**Conclusion:**

These findings indicated that CLB may be used for the treatment of anxiety and improvement of the rank score via regulation of gut microbiota and anti‐inflammatory effects.

## Graphical abstract

These findings indicated that the combination of living Bifidobacterium, Lactobacillus, and Streptococcus may be used for the treatment of anxiety and improvement of the rank score via regulation of gut microbiota and anti‐inflammatory effects.

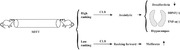

## 1 INTRODUCTION

Lifetime prevalence of anxiety disorders in the population ranges between 5% and 25%, and 12‐month prevalence varies from 3.3% to 20.4% worldwide (Kessler et al., 2009). Anxiety disorders cause a great social burden because of high prevalence and associated disability (Whiteford et al., 2015). Social rank has a strong influence on the behavior and health of humans and animals (Wang et al., 2014). Therefore, the social dominance tube test (SDTT) was used to measure the social rank of mice in the present study. In SDTTs, one mouse will try to acquire dominance and push another animal out of the tube. The dominant mouse wins and gains a higher social rank (Lindzey et al., 1961; Wang et al., 2011). However, the anxiety‐ and depression‐like behaviors of mice after an SDTT have not been reported previously.

Anxiety and depression are stress‐related psychiatric disorders, (Rook & Lowry, 2008) and a fraction of people suffering from depression also manifests immune dysregulation and chronic inflammation (Lindqvist et al., 2017). A hypothesis suggested that in patients or animal models of anxiety and depression, the level of proinflammatory cytokines is increased, inducing a holistic immune response shift toward inflammation. Moreover, psychological stress or disease impairment may induce a dysfunction of the brain–gut axis that results in anxiety and depression (O'Mahony et al., 2011; Scott et al., 2013; Wilhelmsen, 2000). The brain–gut axis is a bidirectional message transformation pathway between the brain and gut in mammals that connects the brain with the gut through several pathways, including nerves and the immune system (Forsythe et al., 2010). The gut microbiota can influence the brain and behaviors through the brain–gut axis, which plays a crucial role in mental disorders, including anxiety and depression (Kelly et al., 2016). Multistrain probiotic treatment (*Lactobacillus helveticus* R0052, *Lactobacillus plantarum* R1012, and *Bifidobacterium longum* R0175) can attenuate anxiety‐ and depression‐like behaviors, increase *Lactobacillus* abundance, and reverse immune changes in the hippocampus (Li et al., 2018). *Lactobacillus helveticus* strain MCC1848 induces resilience to anxiety‐ or depression‐like symptoms attributed to subchronic social defeat stress in mice (Maehata et al., 2019).

However, the impact of a combination of living *Bifidobacterium longum*, *Lactobacillus bulgaricus*, and *Streptococcus thermophilus* (CLB) on the ranking in SDTT has not been reported. The present study demonstrated that CLB plays a powerful positive role in high‐intensity competition. We studied the influence of CLB on anxiety‐ and depression‐like behaviors, social rank, gut microbiota, inflammatory cytokines, and brain‐derived neurotrophic factor (BDNF) after SDTT to explore possible mechanisms related to anti‐inflammatory effects and rebalance of the gut microbiota.

## 2 MATERIALS AND METHODS

### 2.1 Animals

Male 4‐week old ICR (Institute of Cancer Research) mice (body weight: 22 ± 2 g) were acquired from Kunming Medical University. Mice were raised in groups of 4–5 animals per cage (290 mm × 178 mm × 150 mm) in a room with a normal light cycle (22 ± 1°C and 50 ± 2% humidity, 12 h light/12 h dark) and had free access to food and water. The animals were adapted to the laboratory conditions for 1 week before the onset of the experiment. The handling procedures were approved by the Institutional Animal Care and Use Committee of Kunming Medical University and were performed in accordance with the Guide for the Care and Use of Laboratory Animals.

### 2.2 SDTT

SDTT was performed as reported previously with slight modifications (Ward et al., 2013). SDTT is highly transitive and stable and correlates well with other dominance measures. The apparatus was a 30 cm long and 3 cm diameter clear acrylic tube. Two mice were placed at each end of the tube. A mouse will try to exert dominance and flush another animal back out of the tube, and the dominant mouse wins. After cyclic competition, which involved one mouse competing with all other mice, the mice were ranked according to the number of wins from most to least.

### 2.3 Experimental design and sample collection

**FIGURE 1:**
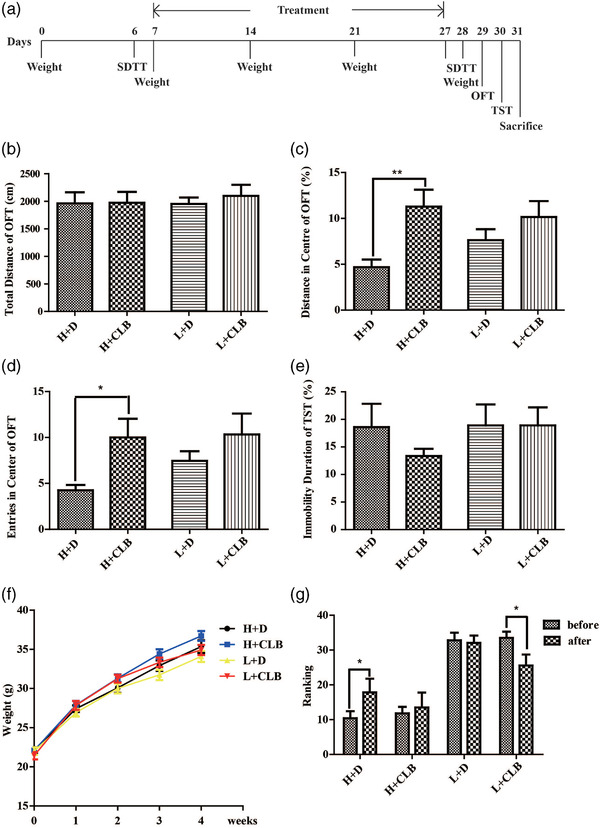
CLB improved the SDTT rank score of low ranking mice and alleviated anxiety‐like behaviors of high ranking mice. (a) Schedule of the experiment. (b) Total distance travelled in the OFT. (c) Distance in the center of the OFT. (d) Entries in the center of the OFT. (e) Immobility duration of the TST. (f) Weight during 5 weeks. (g) Changes in the ranking before and after administration. The data are shown as the mean ± SEM. Significance: * *p* < .05; ** *p* < .01 (*n* = 10–12 per group)

After adapting to the laboratory conditions for 1 week, 45 mice underwent SDTT. High ranking mice (H) included the top 22 mice ranked by a total of 990 SDTTs, and the remaining 23 mice were considered low ranking mice (L). High ranking mice were divided into two groups based on equal rankings: the H+ distilled water (D) group (*n* = 12, received distilled water) and the H+CLB group (*n* = 10, received CLB: 1.25 × 10^7^ CFU/kg *Bifidobacterium longum*, 1.25 × 10^6^ CFU/kg *Lactobacillus bulgaricus*, and 1.25 × 10^6^ CFU/kg *Streptococcus thermophilus*; Inner Mongolia Shuangqi Pharmaceutical Co., Ltd., Hohhot, China). Low ranking mice were allocated to the L+D group (*n* = 11, received distilled water 10 mL/kg) and the L+CLB group (*n* = 12, received CLB) with equal rankings. Mice were given distilled water or CLB (dissolved in distilled water) in the corresponding groups by gavage for 21 days. All mice were subjected to 2 consecutive days of behavioral tests one day after SDTT (Figure 1). On day 31, mice were sacrificed by decapitation after intraperitoneal anesthesia with 6% sodium pentobarbital. The cecal contents were collected and used for 16S rRNA sequence analysis. The whole hippocampus was quickly frozen in liquid nitrogen and stored at −80°C for subsequent western blotting assays.

### 2.4 Open field test

Mice were gently placed in an open field in a box (50 cm × 50 cm ×28 cm) with the floor divided into 25 squares. Nine central squares were defined as the center, and 16 squares along the walls were defined as the periphery. The movements were digitally recorded in a 6‐min trial by SMART video tracking software V3.0 (Panlab, Barcelona, Spain).

### 2.5 Tail suspension test

Mice were suspended upside down by the tail 40 cm above the floor, and adhesive tape was placed at a distance of 1 cm from the tail tip. During a 6‐min testing period, the mice were habituated for the first 2 min, and immobility time was analyzed for subsequent 4 min.

### 2.6 Analysis of diversity of community of intestinal microflora

For 16S rRNA sequence analysis, DNA was extracted and amplified, targeting the V3‐V4 region with indexes and adapter‐linked universal primers 338F (5′‐ACTCCTACGGGAGGCAGCA‐3′) and 806R (5′‐GGACTACHVGGGTWTCTAAT‐3′). The amplicons were extracted, purified and quantified. Purified amplicons were tagged with nucleotide barcodes, pooled in equimolar quantities and paired‐end sequenced on an Illumina MiSeq platform. Raw fastq files were demultiplexed and quality‐filtered by Trimmomatic (Version 3. 29) and then merged by FLASH (version 1.2.7). Operational taxonomic units (OTUs) were obtained with a 97% similarity cutoff, and chimeric sequences were identified and removed by UCHIME. Taxonomy of all 16S rRNA gene sequences was analyzed against the Silva (SSU123) 16S rRNA database, with a confidence threshold of 70%. The data were analyzed via the free online Majorbio I‐Sanger cloud platform (www.i‐sanger.com).

### 2.7 Western blotting of the samples of the hippocampus

The samples of the hippocampus were homogenized in protein extraction reagent containing protease inhibitors, and the concentration of the protein was determined. The proteins were separated by sodium dodecyl sulfate‐polyacrylamide gel electrophoresis using a Mini‐Protean II apparatus (Bio‐Rad, CA, USA). The proteins were electroblotted onto polyvinylidene difluoride membranes, which were blocked. The membranes were incubated with primary antibodies against BDNF (ab108319, 1:1000), interleukin‐1β (IL‐1β, ab9722, 1:1000), ionized calcium binding adapter molecule 1 (Iba1, ab178847, 1:1000) and tumor necrosis factor‐α (TNF‐α, 11948S, 1:1000) overnight at 4°C. The membranes were further incubated with horseradish peroxidase‐conjugated anti‐rabbit IgG (7074S, 1:5000) and developed using ECL reagents. The chemiluminescence signal was imaged by a ChemiDoc XRS system (Bio‐Rad), and the protein band signals were evaluated using ImageJ 1.4.3.67 software. The signals of individual protein bands were normalized to the signal intensity of the β‐actin band and are presented in arbitrary units.

### 2.8 Statistical analysis

**FIGURE 2:**
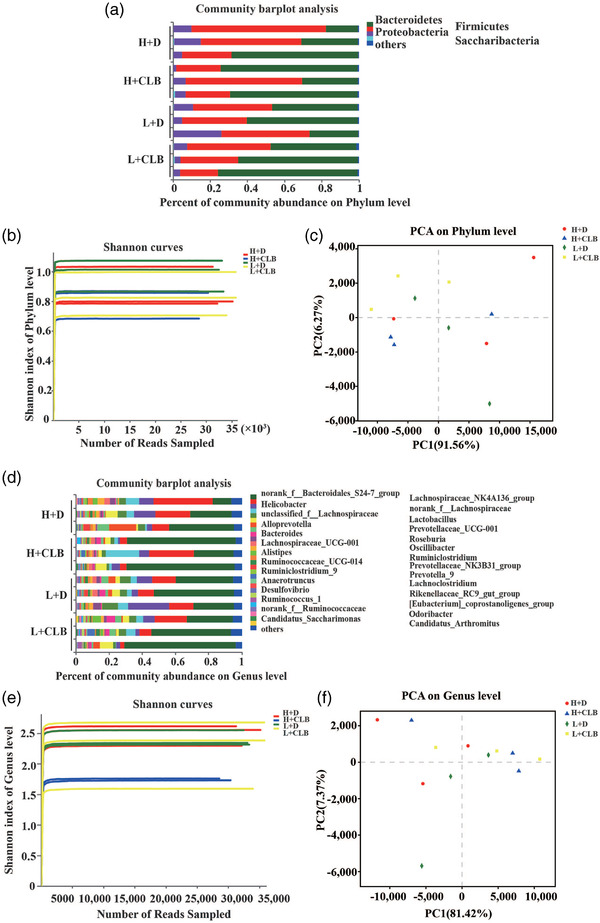
Community bar plot analysis, Shannon index, and PCA. (a) Community bar plot analysis showing the composition of the community and species abundance of the four groups at the phylum level. (b) There were no significant differences in the Shannon index at the phylum level. (c) The PCA results indicated a lack of significant differences in the composition of microbial community at the phylum level. (d) Community bar plot analysis showing the composition of the community and species abundance of the four groups at the genus level. (e) There were no significant differences in the Shannon index at the genus level. (f) The PCA results indicated a lack of significant differences in the composition of microbial community at the genus level (*n* = 3 per group)

The data are expressed as the mean ± SEM and were analyzed using SPSS 19.0 (SPSS Inc., Chicago, IL, USA). *p* Values < .05 were considered statistically significant. The data were analyzed using two‐way ANOVA (status x treatment) when appropriate. The data in Figure 1g were analyzed by paired *t*‐test. The data in Figure 2c and F were analyzed by the Adonis test.

## 3 RESULTS

### 3.1 CLB improved the SDTT rank score of low ranking mice and alleviated anxiety‐like behaviors of high ranking mice

To investigate the influence of CLB on the ranking score and on anxiety‐ and depression‐like behaviors, mice were assigned to four groups, including the H+D, H+CLB, L+D, and L+CLB groups, based on their individual SDTT scores after the first SDTT on day 6. The weight of the animals was measured every week. The second SDTT, OFT, and TST were carried out 3 weeks after the first SDTT (Figure 1a). The results indicated that rank (F_1, 41 _= 0.095, *p* = .759) and CLB (F_1, 41_ = 0.178, *p* = .675) had no effects on spontaneous activity without interaction between these factors (Figure 1b). CLB treatment resulted in a longer distance from the center of the OFT (F_1, 41 _= 10.046, *p* = .003, Figure 1c), and CLB was the major anxiolytic factor; however, rank (F_1, 41 _= 0.393, *p* = .534) or interaction between these factors had no effect. CLB treatment resulted in a higher number of entries in the center of the OFT (F_1, 41 _= 4.857, *p* = .033, Figure 1d), and CLB was the major anxiolytic factor; however, rank (F_1, 41 _= 1.825, *p* = .184) or interaction between these factors had no effect. CLB (F_1, 41 _= 0.068, *p* = .796) or rank (F_1, 41 _= 0.394, *p* = .534) did not influenced the immobility duration without interaction between these factors (Figure 1e). The weight of all animals was steadily increased with time (Figure 1f). The rank score of H+D mice in the second SDTT was lower (an increase in rank number) than that in the first SDTT (paired *t*‐test, *p* < .05), and L+CLB mice acquired a considerably higher rank (a decrease in rank number) after CLB treatment (paired *t*‐test, *p* < .05; Figure 1g), indicating that CLB treatment increased the rank score of the losers. Thus, CLB improved the SDTT ranking score of low ranking mice and alleviated anxiety‐like behaviours of high ranking mice.

### 3.2 CLB decreased the level of *Desulfovibrio* and increased the level of *Mollicutes* in the feces

To investigate whether CLB and the rank of mice influence the composition of microbial community, 16S rRNA sequence analysis was performed in fecal samples. The results of community bar plot analysis illustrated the community composition and species abundance in the four groups at the phylum level (Figure 2a). CLB (F_1, 8_ = 2.114, *p* = .184), rank (F_1, 8_ = 1.092, *p* = .327) or their interaction did not influence the Shannon index (Figure 2b), and there were no differences in PCA (R^2 ^= 0.262, *p* = .457, Figure 2c) at the phylum level. At the genus level, the community composition and species abundance were determined by community bar plot analysis (Figure 2d). CLB (F_1, 8 _= 3.471, *p* = .099), rank (F_1, 8 _= 0.244, *p* = .635), or their interaction did not influence the Shannon index (Figure 2e), and there were no differences in PCA (R^2 ^= 0.306, *p* = .373, Figure 2f), indicating that there were no significant differences in the composition of microbial community of the four groups at the phylum or genus levels.

**FIGURE 3:**
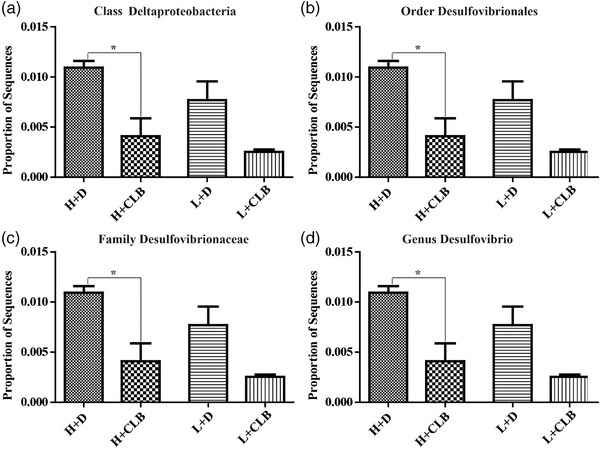
CLB decreased the level of *Desulfovibrio* in the feces. The proportion of the sequences of (a) *Deltaproteobacteria* at the class level, (b) *Desulfovibrionales* at the order level, (c) *Desulfovibrionaceae* at the family level, and (d) *Desulfovibrio* at the genus level. The data are shown as the mean ± SEM. Significance: * *p* < .05 (*n* = 3 per group)

The results of subsequent analysis indicated that CLB treatment decreased the proportion of sequences of Deltaproteobacteria at the class level, Desulfovibrionales at the order level, Desulfovibrionaceae at the family level, and *Desulfovibrio* at the genus level, and CLB was the major factor (F_1, 8 _= 15.936, *p* = .004, Figure 3a–d). However, without interaction, the rank did not influence the proportion of sequences of Deltaproteobacteria at the class level, Desulfovibrionales at the order level, Desulfovibrionaceae at the family level, or *Desulfovibrio* at the genus level (F_1, 8 _= 1.595, *p* = .242, Figure 3a–d). These results indicated that CLB decreased the level of *Desulfovibrio* in the feces.

**FIGURE 4:**
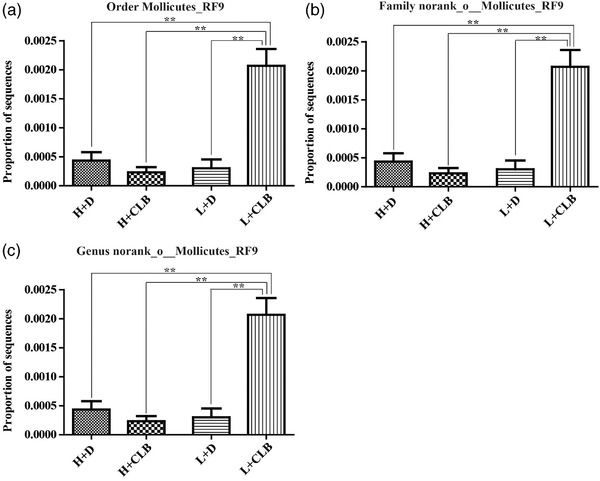
CLB increased the level of *Mollicutes* in the feces. The proportion of the sequences of (a) *Mollicutes_RF9* at the order level, (b) *norank_o__Mollicutes_RF9* at the family level, and (c) *norank_o__Mollicutes_RF9* at the genus level. The data are shown as the mean ± SEM. Significance: ** *p* < .01 (*n* = 3 per group)

The results indicated that CLB treatment increased the proportion of sequences of Mollicutes_RF9 at the order level and norank_o_Mollicutes_RF9 at the family and genus levels, and the effects of CLB (F_1, 8 _= 17.959, *p* = .003), rank (F_1, 8 _= 21.146, *p* = .002), and interaction between these factors were significant (F_1, 8 _= 28.301, *p* = .001, Figure 4a–c).

The datasets of 16S rRNA sequencing generated in the present study can be found at https://www.ncbi.nlm.nih.gov/sra/PRJNA524444.

### 3.3 CLB increased the expression of BDNF and decreased the expression of TNF‐α in the hippocampus

**FIGURE 5:**
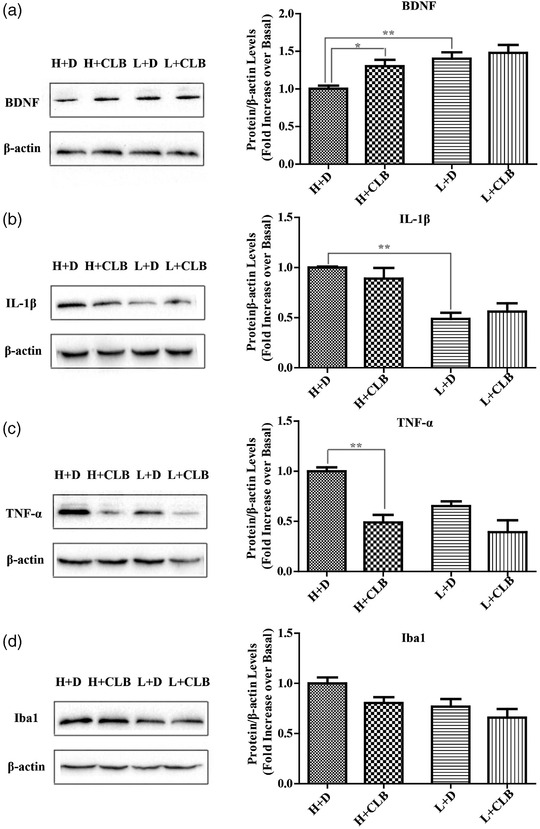
CLB increased the level of BDNF and decreased the level of TNF‐α in the hippocampus. Protein levels of (a) BDNF, (b) IL‐lβ, (c) TNF‐α, and (d) Iba1. The data are shown as the mean ± SEM. Significance: * *p* < .05; ** *p* < .01 (*n* = 4 per group)

We measured the expression of several inflammatory cytokines (Iba1, IL‐lβ and TNF‐α) and neurotrophic factor (BDNF) in the hippocampus of mice. CLB treatment induced an increase in BDNF, and both CLB (F_1, 12 _= 5.186, *p* = .042) and rank had an effect (F_1, 12 _= 12.104, *p* = .005, Figure 5a); however, interaction between these factors had no effect. CLB treatment had no effect on IL‐lβ (F_1, 12 _= 0.064, *p* = .805), and rank had an effect (F_1, 12 _= 31.637, *p* = .000, Figure 5b); however, interaction between these factors had no effect. CLB treatment induced a significant decrease in TNF‐α (F_1, 12 _= 16.701, *p* = .002), and the rank (F_1, 12 _= 9.341, *p* = 0.1, Figure 5c) or interaction between rank and CLB treatment had no effects. CLB treatment had no effects on Iba1 (F_1, 12 _= 4.685, *p* = 0.051, Figure 5d), and rank had an effect (F_1, 12 _= 7.127, *p* = 0.20); interaction between these factors had no effect.

## 4 DISCUSSION

To determine the influence of CLB on the ranking score and anxiety‐ or depression‐like behaviors, mice after the first SDTT were fed CLB. The results indicated that CLB improved the SDTT ranking score of the losers and alleviated anxiety‐like behaviors of the winners. CLB regulated the intestinal flora, increased the expression of BDNF, and decreased the level of TNF‐α in the hippocampus.

In our study, the IL‐lβ expression in the H+D group was higher than that of the L+D group after the high‐intensity competition, which may be due to the sustained chronic stress (Fan et al., 2018) caused by a high frequency of wins.

CLB contains three probiotics: *Bifidobacterium longum*, *Lactobacillus bulgaricus*, and *Streptococcus thermophilus*. The results of the present study are supported by a study that demonstrates probiotic *Bifidobacterium longum* alleviates immobilization stress‐induced anxiety in mice (Han & Kim, 2019). Anxiolytic effect of *Bifidobacterium longum* is attributed to the suppression of gut dysbiosis, mitigation of the activation of the hippocampal transcription factor nuclear factor kappa‐B (NF‐κB), and a reduction in blood levels of corticosterone, TNF‐α, IL‐6, and lipopolysaccharide (Han & Kim, 2019). The expression of TNF‐α in the CLB‐treated group in the present study was significantly lower than that in the distilled water‐treated group after high‐intensity competition, which may be due to anti‐inflammatory effects of *Bifidobacterium* species manifested as a decrease in the TNF‐α level (Klemenak et al., 2015; Primec et al., 2018). Additionally, *Lactobacillus* strains reduced the level of TNF‐α in an LPS‐stimulated mouse model (Reyes‐Díaz et al., 2018). *Streptococcus thermophiles* is a new promising probiotic candidate that promotes the recovery in mice with antibiotic‐associated diarrhea and reduces the levels of proinflammatory cytokines (TNF‐α) (Hu et al., 2020). These results confirm that anti‐inflammatory effect of CLB is due to a reduction in the level of TNF‐α. TNF‐α induces the development of anxiety‐like behavior in normal mice (André et al., 2014). A blockade of TNF‐α has also been shown to reduce anxiety‐like behavior and inflammation in mice (Chen et al., 2013). TNF‐α plays a critical role in pathogen‐associated molecular patterns acting on the cell surface receptors, such as Toll‐like receptors (TLRs), to mediate downstream signaling that culminates in NF‐κB stimulation (Baker et al., 2011). Experimental animals subjected to chronic stress manifested the activation of NF‐κB mediated by upregulation of TLR4, leading to neuroinflammation, oxidative stress, anxiety, and depressive‐like behaviors (Gong et al., 2018). Thus, TLR4/NF‐κB is a major signaling pathway involved in the pathogenic mechanisms of anxiety and represents a novel direction for our future studies.

The results of the present study indicated that CLB treatment decreased the abundance of Deltaproteobacteria, Desulfovibrionales, Desulfovibrionaceae, and *Desulfovibrio*, and CLB played an anxiolytic role, which was consistent with the effect of chlorogenic acid, an antidepressant ingredient, in a rat model of major depression disorder (Song et al., 2019). Notably, a strong link between *Desulfovibrio* and inflammation has been demonstrated. The degree of mucosal inflammation may be correlated with the *Desulfovibrio* burden (Lennon et al., 2014). The plasma concentration of TNF‐α is reduced in mice fed a prebiotic due to a reduction in the level of *Desulfovibrio* and a decrease in inflammatory parameters (Sawin et al., 2015). Moreover, antiageing and antioxidant effects are accompanied by the regulation of the abundance of *Desulfovibrio*, a decrease in TNF‐α, an increase in BDNF, and protection of hippocampal neurons (Liu et al., 2019). Furthermore, *Desulfovibrio* can be involved in the pathogenesis of periodontal inflammation through stimulation of human gingival fibroblasts (Dzierzewicz et al., 2010). These studies provide evidence on the anti‐inflammatory effects of CLB associated with a reduction in the level of *Desulfovibrio*.

The data on gut microbiota indicated that CLB increased the abundance of Mollicutes_RF9 in loser mice, and the ranking score of the CLB+L group demonstrated a most pronounced increase. The data indicated that advanced ranking score may be related to the highest abundance of Mollicutes; however, additional studies are needed to confirm a correlation between the two factors. Direct relationships between Mollicutes and probiotics have not been reported in the literature; however, a study demonstrated a significant reduction in the relative abundance of Mollicutes in MDD patients (Zhu et al., 2019). (R)‐Ketamine, an antidepressant, significantly increases the abundance of Mollicutes at the class level in susceptible mice (Yang et al., 2017), indicating that an impact of CLB on Mollicutes is similar to the effect of (R)‐ketamine. Antidepressant treatment in rodents results in competitive behavior in submissive mice (Malatynska et al., 2005). These results may explain a possible role of Mollicutes in the effects of CLB; however, the physiological mechanism of action of Mollicutes in the regulation of the ranking score is unclear. This hypothesis requires confirmation by additional experiments.

The results of the present study indicated that the level of BDNF, a neurobiological marker for anxiety (JiaWen et al., 2018), was increased in response to CLB treatment, which had an anxiolytic effect on behaviors and decreased the level of TNF‐α in the hippocampus in winning mice. Ingestion of *Bifidobacterium longum* regulates emotional behavior and the central BDNF pathway in chronic stress mice by reshaping the gut microbiota (Tian et al., 2019). Augmentation of BDNF is involved in anti‐inflammatory effects in the hippocampus (Zhang et al., 2016). Adult neurogenesis in the hippocampus is modulated by specific microglia groups and functionally involved in behavioral responses to stress. IL4‐driven microglia, characterized by high expression of Arg1, in the hippocampus trigger BDNF‐dependent neurogenesis, which is a response to chronic stress and a protection against depression‐like behaviors (Zhang et al., 2021). The inflammatory responses contribute to neurogenesis impairments (Han et al., 2020), dysregulation of tryptophan‐kynurenine metabolism (Murray et al., 2015), and dysfunction of the HPA axis (Zhao et al., 2017), which is associated with the depression‐like behaviours. Inflammatory responses mediated by microglia lead to stress‐induced neurodevelopmental deficits. Peroxisome proliferator‐activated receptor gamma activation is one of the reasons of microglial activation phenotypes (Han et al., 2020). Changes in brain function induced by systemic inflammation are related to Type I interferons activity, full expression of the IL‐6 response, and activation of the tryptophan‐kynurenine metabolism pathway (Murray et al., 2015). Additionally, stress‐induced dysfunction of the hypothalamic‐pituitary‐adrenal (HPA) axis is associated with the occurrence of mood disorders. A heightened neuro‐inflammatory response and oxidative stress in mouse hypothalamus play a fundamental role in HPA axis disorders and depressive behaviors (Zhao et al., 2017). Moreover, the activation of BDNF reduces inflammation via TNF‐α in an injury model in rats (Liang et al., 2019). Various alterations in structural synaptic plasticity in hippocampal neurons are mediated by overproduction of TNF‐α. The complexity of excitatory synaptic connectivity and BDNF expression are reduced in the hippocampus after peripheral nerve injury, and the opposite changes depend on TNF‐α receptor 1 signaling (Liu et al., 2017).

## 5 CONCLUSION

**FIGURE 6:**
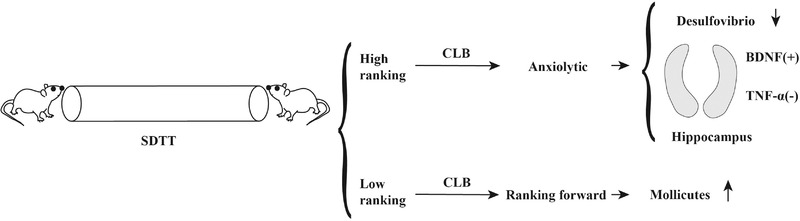
Mechanisms of the effect of CLB on the ranking score and anxiety‐like behaviors

In conclusion, the results of the present study suggested that CLB improved the SDTT ranking score of the losers and alleviated anxiety‐like behaviors of the winners. Moreover, CLB decreased the level of *Desulfovibrio* and augmented the level of Mollicutes in the feces, increased the level of BDNF, and reduced the level of TNF‐α in the hippocampus (Figure 6). The data of the present study suggest that administration of CLB regulates social dominance and anxiety‐like behaviors. The findings of the present study indicated that CLB may have potential applications in the treatment of anxiety and for improvement of rank score through regulation of the gut microbiota and anti‐inflammatory effects.

## CONFLICT OF INTEREST

The authors declare no conflict of interests.

### PEER REVIEW

The peer review history for this article is available at https://publons.com/publon/10.1002/brb3.2453

## Data Availability

The analyzed data sets generated during the study are available from the corresponding author on reasonable request.
